# Work‐Life Perceptions of Danish Personal Care Assistants Working in the Home Mechanical Ventilation Setting—A Survey Study

**DOI:** 10.1111/scs.70098

**Published:** 2025-08-21

**Authors:** Pernille Bang Nielsen, Anette Bjerregaard Alrø, Anna Holm, Dorthe Enggaard Trojel, Pia Dreyer

**Affiliations:** ^1^ Department of Intensive Care Aarhus University Hospital Aarhus N Denmark; ^2^ Section of Nursing, Institute of Public Health Aarhus University Aarhus C Denmark

**Keywords:** home mechanical ventilation, personal care assistants, survey study, training, work environment, work‐life

## Abstract

**Background:**

Working as a personal care assistant (PCA) in the homes of mechanical ventilation users can be challenging due to the job's complexity. Research indicates that a poor work environment among PCAs could lead to risks of resignations and a high staff turnover, potentially affecting healthcare quality in home mechanical ventilation (HMV) setups.

**Aim:**

To explore work‐life perceptions of Danish PCAs working in HMV settings, with a specific focus on communication quality, physical and psychological work environment and the PCA training programme.

**Methods:**

PCAs affiliated with a respiratory centre in Denmark were surveyed via an online questionnaire between June and September 2022. Quantitative data from 460 PCAs were analysed using descriptive statistics, whereas qualitative data from free‐text responses was examined through content analysis. The Consensus‐Based Checklist for Reporting of Survey Studies (CROSS) was used as a guideline.

**Results:**

PCAs generally rated their work‐life positively. Qualitative findings revealed challenges in communication and collaboration with the HMV users and their families and formal actors from whom PCAs sought support. Working alone, combined with a strong sense of responsibility for the needs of users and their families, imposed a significant mental burden on PCAs, further exacerbated by the fact that they felt left to themselves in challenging situations. Although PCAs expressed overall satisfaction with training in respiratory care and monitoring, they highlighted the need for a more comprehensive training programme to better equip them for their roles.

**Conclusion:**

PCAs experienced a strong sense of responsibility for providing high‐quality healthcare for HMV users and their families, which mentally burdened them. They emphasised the need for improved organisational support and training to effectively manage the diverse and complex situations they encountered in the home setting.

## 1 Introduction

In recent decades, long‐term life support technologies, including mechanical ventilators, have become well‐established treatments in Western countries, significantly prolonging survival for people with chronic respiratory insufficiency [1, 2, 3, 4, 5]. Mechanical ventilation can be administered invasively via tracheostomy or noninvasively via a mask, for some hours a day or continuously. This treatment is often delivered in a home setting, commonly referred to in the literature as home mechanical ventilation (HMV). Home‐based treatment reduces hospital admissions [6], and HMV allows users to stay in their family home despite substantial care needs. The organisation and provision of care for HMV users varies across countries [7, 8]; however, in many countries, family members assume this responsibility [9, 10, 11]. In Denmark, users are supported by personal care assistants (PCAs) who provide respiratory care and monitoring within the family home [12, 13].

HMV setups include various formal and informal actors, making their organisational structure quite complex [6, 14]. In Denmark, HMV treatment is prescribed by a respiratory centre and delegated to PCAs, who have completed a formal HMV training programme. PCA arrangements vary significantly due to differences in legal agreements, employment types and professional requirements [15]. In most countries, HMV setups are costly for society and constitute a significant component of overall health expenditure [16, 17, 18]. This is also the case in Denmark, where HMV setups, including PCA arrangements, are financed through the welfare system [15].

HMV requires a demanding advanced home care programme [12, 19]. Users and their families rely on PCAs for respiratory care and monitoring [6, 12, 14] and providing HMV care through the use of PCAs has the potential to support everyday family life from a patient‐ and family‐centred care (PFCC) approach [20]. However, the presence of PCAs in the home can impose a burden, affecting the user and family physically, emotionally, socially and existentially [6, 21, 22, 23, 24]. Often, the home must be modified to meet hygiene and ergonomic standards suitable for a working environment, which can make it resemble a hospital setting [12]. The private sphere is challenged when PCAs enter the home, which may lead to feelings of being invaded, leaving the user and family without a private space to be themselves [13, 25, 26, 27].

Although PCA arrangements have existed in Denmark and a few other countries for some decades, research on the PCA perspective remains sparse. Existing studies highlight several areas that require further investigation. Lindahl and Kirk [12] emphasise the need for PCAs and families to develop a professional and stable relationship, as their cooperation is often long‐term. Still, some PCAs experience difficulties in their communication and relationships with the user and family. Some feel that their presence is hardly accepted or, on the contrary, that they are treated as a private friend [28, 29]. The family's personal experience with HMV treatment and the underlying disease can make them take on a role as ‘experts’. Thus, they may have specific attitudes about how the treatment should be handled, demanding PCAs to adapt to family care standards and, therefore, balance being a professional carer and a respectful guest in the home [30]. Such dynamics can lead to role confusion, conflicts and power struggles [12, 14, 29, 30]. Despite having completed HMV training programmes, PCAs still experience a great sense of responsibility in handling HMV treatment and various care needs in relation to this, which keeps them constantly alert and can lead to mental exhaustion [14, 28]. They typically work alone with no or limited access to communication and support from formal actors in the HMV setup, contributing to feelings of insecurity and work‐life loneliness [28, 29, 30]. Furthermore, research has identified inadequate physical working conditions, including insufficient space and poor safety measures [12, 14, 28].

The sparse amount of research available indicates various day‐to‐day work‐life challenges faced by PCAs in these highly complex HMV settings, revealing a critical knowledge gap that needs to be addressed to prevent potential consequences. A poor work environment amongst PCAs can lead to risks of resignations, team breakdowns and high staff turnover [12, 29]. This, in turn, may result in a lack of continuity in care, reduced healthcare quality for the HMV user and their family, along with increased health care costs for the welfare system such as expenses for training new PCAs and admission to intensive care units [19, 28]. Therefore, understanding the work‐life perceptions of PCAs is paramount to improve and foster a sustainable work‐life for these highly important frontline actors, ultimately ensuring stable PCA arrangements and the provision of high‐quality HMV for users and their families.

## 2 Aim

This study aimed to explore work‐life perceptions of Danish PCAs working in the HMV setting, with a specific focus on communication quality within the HMV setup, the physical and psychological work environment and the quality of the PCA training programme.

## 3 Methods

### 3.1 Study Design

A web‐based descriptive survey study was conducted amongst PCAs affiliated with a respiratory centre at a Danish University Hospital. This design was used to reach the wide group of PCAs working in the HMV setting. The Consensus‐Based Checklist for Reporting of Survey Studies (CROSS) was used to ensure explicit and appropriate reporting of the study (Appendix S1) [31].

### 3.2 The Danish HMV Setting

Denmark has three respiratory centres that prescribe and delegate HMV treatment to PCAs employed under legally established PCA arrangements. In May 2024, there were 522 HMV users with PCA arrangements in Denmark, including both children and adults. PCAs in Denmark form a heterogeneous group of people with varying levels of education, ranging from no formal health training to qualifications as healthcare professionals, including social and healthcare assistants, social and healthcare helpers and nurses.

PCAs' responsibilities are based on user‐specific treatment guidelines developed by the respiratory centres, which are also responsible for providing training in respiratory care and monitoring. Training of a new PCA team is conducted at the respiratory centres, whereas training of individual new PCAs occurs in the user's home through peer‐to‐peer instruction. The respiratory centres also provide a hotline service, enabling access to HMV specialists from the in‐home setting.

PCA arrangements vary depending on the underlying legal agreements and are therefore organised in various ways. Some arrangements involve employment agencies contracting PCAs, whereas others have PCAs employed directly by the municipality or the users themselves, who formally assume the role of employer and supervisor. In addition to respiratory care needs, most HMV users have extensive personal care needs due to their diagnosis. These needs are assessed and funded by the municipality, separately from respiratory care needs. However, they are often provided by the same PCAs under a shared assistance arrangement between the regionally managed respiratory centre and the municipality [32].

### 3.3 Participants

The study included PCAs working with both paediatric and adult HMV users. All HMV users have chronic respiratory insufficiency, although the underlying medical causes are quite diverse. In this study, we categorised these causes into three broad disease groups:

1. Neuromuscular diseases such as muscular dystrophy (e.g., Duchenne, Becker and SMA).
2. Neurodegenerative diseases such as amyotrophic lateral sclerosis (ALS).
3. Other health issues causing a need for HMV (e.g., tetraplegia, apoplexies and rare diseases).

No exclusion criteria were established, as the objective was to ensure variation through a broad inclusion of PCAs. Potential study participants (*n* = 1619) were identified through an electronic system used to register affiliated PCAs at the respiratory centre. All participants who were registered at the time of the study were included.

### 3.4 Data Collection and Procedure

The questionnaire was developed based on themes identified in the presented literature on the PCA perspective, as well as clinical knowledge from the respiratory centre. Since some of the research presented in this article was conducted locally at the respiratory centre [13, 24, 27, 28], the findings from this research naturally served as the foundation for the establishment of the study's aim and were subsequently incorporated into the questionnaire development process. Our objective was to ask questions that addressed various aspects of PCAs' work‐life challenges, including themes such as demographics, communication with various actors in the HMV setup, work environment and training. Questions on work environment were guided by the definition of a healthy work environment established by WHO [33]. Examples of questionnaire items, questions and response options are presented in Table 1.

**TABLE 1:** Examples of questionnaire items, questions and response options.

Items	Questions	Response options
Communication quality with user, family, employer and respiratory centre	How is your communication with your employer? Is there anything you would like to elaborate on regarding your communication with your employer?	Rating on Likert scale (good, acceptable, poor, very poor, irrelevant) Open text field
Work environment, physical and psychological	How satisfied are you with your psychological work environment? Is there anything you would like to elaborate on regarding your psychological work environment?	Rating on Likert scale (Very satisfied, satisfied, somewhat satisfied, neither satisfied nor dissatisfied, somewhat dissatisfied, dissatisfied, very dissatisfied) Open text field

The online survey tool *SurveyXact* [34] was used for questionnaire design. Before distribution, the questionnaire was validated through pilot testing performed by specialists from the respiratory centre (*n* = 5) and affiliated PCAs (*n* = 2).

An email was sent to 1619 PCAs in June 2022, including information about the purpose of the survey and a weblink to the questionnaire for individual completion. The exact number of eligible informants among the 1619 recipients was unclear due to outdated employment records. As this couldn't be resolved before distributing the questionnaire, the response rate could not be calculated. Data were collected through *SurveyXact* until the end of September 2022. During this period of time, a reminder was sent to increase the number of responses.

### 3.5 Data Analysis

Only fully answered questionnaires were included for analysis. Quantitative data analysis was conducted using Stata (version 17). Descriptive statistics were used to analyse data, including frequency and percentages. The questionnaire included open‐ended questions, resulting in the collection of a large amount of qualitative data. PCAs demonstrated a persistent need to provide detailed descriptions, which was duly considered during the data analysis. Qualitative data analysis was performed with inspiration from Elo and Kyngäs' [35] description of inductive content analysis. The analysis consists of open coding, grouping of data, categorisation and, lastly, abstraction through which the general categories were formed [35]. In accordance with Elo and Kyngäs' [35] approach and their guidelines for ensuring methodological quality, appropriate measures have been implemented to ensure rigour and trustworthiness; throughout the analysis, credibility was ensured by systematic coding and regular debriefing in the research team, whereas transferability was supported through detailed contextual descriptions. Dependability and confirmability were strengthened by maintaining a clear audit trail and reflexive documentation throughout the process.

### 3.6 Ethical Considerations

According to Danish law, questionnaire studies of this type do not require formal ethical approval from the regional ethics committee [36]. The study adhered to the principles outlined in the Declaration of Helsinki [37] and the Ethical guidelines for nursing research in the Nordic countries [38]. The department's management approved the study and the distribution of the questionnaire. Participants were informed of the study in a written email, including information on data anonymisation, and participation was considered tantamount to study consent. All data were anonymised, stored and analysed within secured hospital systems, ensuring that identification was not possible.

## 4 Results

### 4.1 Sample

The questionnaire was answered by 600 PCAs, of whom 460 completed the questionnaire in its entirety and were therefore included in the final sample (*n* = 460). The characteristics of survey respondents are presented in Table 2.

**TABLE 2:** Characteristics of survey respondents (*n* = 460).

Characteristics	*n* (%)
Age group	
18–27 years	24 (5%)
28–37 years	58 (13%)
38–47 years	103 (22%)
48–57 years	163 (36%)
58–67 years	112 (24%)
Sex	
Female	392 (85%)
Male	68 (15%)
Level of education	
PCA with no formal health training	158 (34%)
Social and health care helper	70 (15%)
Social and health care assistant	186 (41%)
Nurse	12 (3%)
Student	5 (1%)
Other	29 (6%)
Number of years of experience as a PCA	
0–1 years	149 (32%)
2–5 years	168 (37%)
6–10 years	82 (18%)
11–20 years	51 (11%)
21–30 years	10 (2%)
Type of employer	
Employed by employment agency	247 (54%)
Employed by the user	162 (35%)
Employed through an alternative municipal arrangement	51 (11%)
Type of employment	
Permanently employed	240 (52%)
Hourly employed	220 (48%)
Number of years of employment at the current user	
0–1 years	217 (47%)
2–5 years	162 (35%)
6–10 years	26 (6%)
11–20 years	12 (3%)
> 20 years	6 (1%)
Acute temporary worker (no regular user)	37 (8%)
PCAs relation to the current user	
Professional relation	452 (98%)
Family relation	8 (2%)
Age group of current user	
Child (≤ 17 years)	36 (8%)
Adult (≥ 18 years)	424 (92%)
Overall disease category of current user	
ALS	159 (35%)
Neuromuscular diseases	152 (33%)
Other health issues causing a need for HMV	149 (32%)

### 4.2 Communication

**FIGURE 1:**
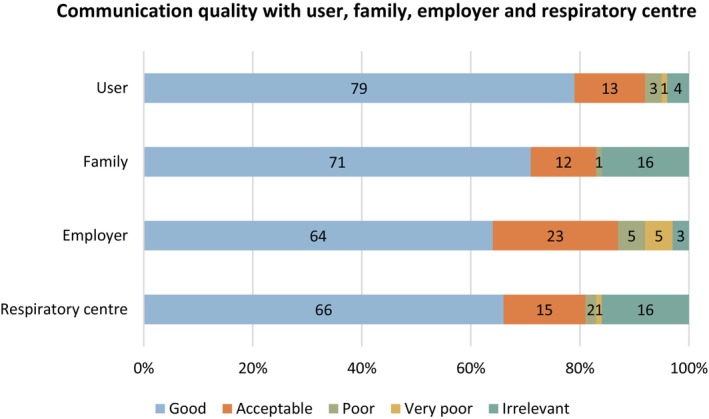
PCAs' perception of communication quality with user, family, employer and respiratory centre.

Figure 1 illustrates PCAs' perception of the quality of communication with the user, family, employer and respiratory centre. In all four categories, the majority of PCAs rated communication positively, with scores ranging from 64% to 79%.

#### 4.2.1 Communication With the User

Overall, respondents found communication with the user good (79%). The qualitative data illustrated that some PCAs found communication challenging when the disease or the HMV treatment meant that the user had no or limited verbal language: ‘The user is so very affected by his disease, ALS, that communication is quite poor. We use a yes/no sign to clarify closed‐ended questions’.

#### 4.2.2 Communication With the Family

Most PCAs found communication with the user's family good (71%). The qualitative findings illustrated a diversity in how PCAs perceived communication with the family. Several PCAs found communication well‐functioning: ‘I have always liked working with that family’. However, some PCAs had experienced considerable challenges in this regard. One PCA described how the family did not seem to acknowledge the PCAs' presence in the home: ‘His family isn't very welcoming… The father won't say hello when we walk in the door and they do not talk to us at the table’. Other PCAs experienced that the family would express scepticism and doubts about their competencies: ‘The family often looks at the PCA team with sceptical eyes when unexpected situations occur’.

#### 4.2.3 Communication With the Employer

Communication with the employer was rated as good by 64%, acceptable by 23% and poor or very poor by 10%. Some qualitative findings underpin the less positive ratings as they point to challenges in communication between the PCAs and the employer. A considerable group of PCAs working via employment agencies expressed a need for support from their employment consultant. However, they found it hard to establish contact: ‘My employer doesn't answer when I contact them…’ Thus, several PCAs felt a lack of support related to the work environment and in‐home collaboration. Furthermore, PCAs experienced poor communication from the employment consultants when changes in care initiatives or work arrangements had to be conveyed to the PCA team: ‘We don't receive any information from our employer; most often, we are the ones who have to communicate everything with the family and each other…’

#### 4.2.4 Communication With the Respiratory Centre

Communication with HCPs from the respiratory centre was rated as good by 66% of respondents, acceptable by 15% and poor or very poor by 3%. The qualitative findings illustrated diverse perceptions of communication with the respiratory centre. Many respondents found support from HCPs helpful, whereas others described experiences of them being busy and stressed, which could result in less supportive communication: ‘The respiratory centre doesn't take my reflections on the user's condition seriously, despite it seeming critical. For example, when my user lacks oxygen because of leakage from the tracheostomy, and I have heard this quite clearly, it is shut down as a problem that doesn't exist’. Other PCAs emphasised the need to be able to ‘…ask stupid questions…’ to learn from challenging situations in the home setting.

### 4.3 Satisfaction With Job Overall and Work Environment

**FIGURE 2:**
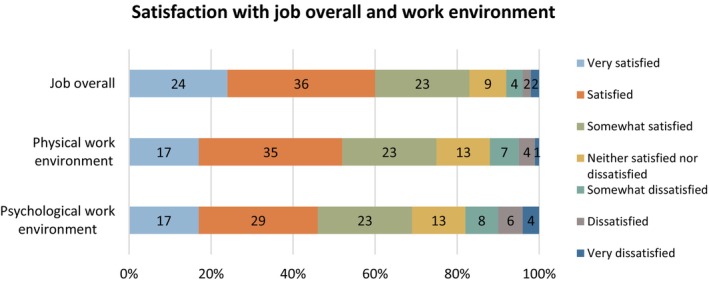
PCAs' satisfaction with job overall and work environment.

Figure 2 illustrates PCAs' satisfaction with their job overall and their satisfaction with the physical and psychological work environment. The majority of respondents were very satisfied, satisfied or somewhat satisfied with their job overall (83%), their physical work environment (75%) and their psychological work environment (69%).

#### 4.3.1 The Job Overall

The qualitative findings provided insights into PCAs' job satisfaction, which largely depended on their ability to thrive under the general working conditions. As PCAs work independently without daily colleagues, some appreciated this aspect of the job: ‘I feel really good in my own company, so the job fits me well’, ‘I love the 1:1 care and calmness'. Others, however, found it isolating: ‘It is very lonely’. In addition, working long shifts (12–24 h) and always having to be available for the user could affect work satisfaction: ‘No breaks during a twelve‐hour shift can be challenging’.

#### 4.3.2 Physical Work Environment

Although 75% of PCAs reported that the physical work environment was satisfactory, some highlighted challenges associated with working in the home setting, including limited space, insufficient assistive equipment, poor indoor climate, heavy lifting and awkward work postures: ‘It's difficult to get good working conditions in the home… No one takes responsibility for our working posture, whether it's poor chairs, sloping walls with little space, high indoor temperatures…’ As a result, several PCAs believed they were at risk of workplace injuries.

#### 4.3.3 Psychological Work Environment

Concerning the psychological work environment, 18% of PCAs reported being somewhat dissatisfied, dissatisfied or very dissatisfied. The qualitative data revealed varied perceptions of the psychological work environment. Some were very satisfied: ‘In general, we have a great psychological work environment’, whereas others were not: ‘It is a very poor psychological work environment…’

Many PCAs found the responsibility of providing life‐sustaining respiratory care and monitoring as overwhelming: ‘I felt a heavy responsibility, and I was afraid to be put in a difficult emergency situation that I wouldn't be able to handle… I didn't thrive in this, had stomach aches, and had to quit the job’. A significant factor affecting the psychological work environment was the user's behaviour. Some PCAs faced challenging and unpredictable behaviour due to the individual user's mental or cognitive state: ‘It is stressful when the user takes out their frustrations on the staff. And at the same time, you have to be sympathetic and offer comfort’. The family's response to their life situation could also affect the PCA: ‘When you work in a home with a user with ALS, the family is very affected by this. Sometimes it takes up all the space, and then it can be tough to get through the shifts’. One PCA described how she felt responsible for supporting the family: ‘The family is in a crisis, which also has to be dealt with…’

Finally, several PCAs experienced a general lack of organisational support, which made them feel left to themselves: ‘The way I see it, the user has a big and supportive network, but that's in no way the case for the PCA’. ‘We are alone at the workplace, with no witnesses, no one to take over in difficult situations and no one to support you and back you up in your views of the situation’.

### 4.4 Training Programme

**FIGURE 3:**
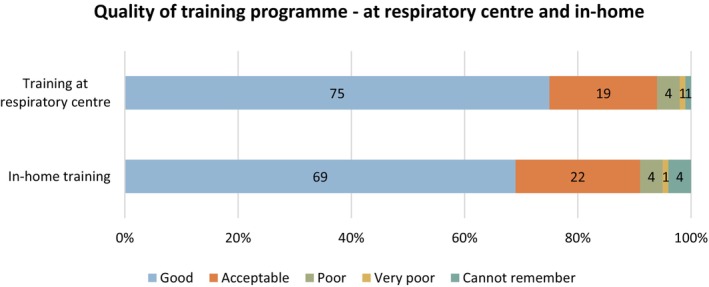
PCAs' perception of the training programme's quality at the respiratory centre and in‐home.

Figure 3 illustrates PCAs' perceptions of the training programme completed at the start of their employment, either at the respiratory centre or in the user's home by PCA colleagues. Most respondents rated the training programme at the respiratory centre as good (75%); while 19% found it acceptable. In comparison, 69% of respondents found the in‐home training programme good; while 22% found it acceptable.

#### 4.4.1 Training at the Respiratory Centre

Several PCAs expressed satisfaction with the training programme at the respiratory centre: ‘I've experienced really good support and fantastic training at the respiratory centre…’ However, some experienced a lack of consistency among the HCPs in their approach to training and evaluation of competencies: ‘They have to work on ensuring consistency in the training process. Having completed several training programmes, I've experienced several differences in the individual nurses’ interpretation of the existing guidelines. Thus, sometimes you end up in the crossfire between two nurses, where one will approve your work and the other won't’.

#### 4.4.2 In‐Home Training

Despite the predominantly positive ratings of the in‐home training programme, a group of PCAs expressed scepticism about it. Several PCAs had experienced a lack of competencies among those responsible for delivering the in‐home training: ‘Peer‐to‐peer training isn't always good… You often experience PCAs who don't have the competencies to teach others’. Additionally, these PCAs described a work culture in which colleagues chose actions based on personal convenience or belief rather than adhering to formal HMV guidelines: ‘So much bad peer‐to‐peer training occurs, where unbelievably many poor and self‐invented rules and approaches are passed on…’ Another respondent elaborated: ‘…you have to be strong to resist that culture’. As a result, some PCAs believed that in‐home training should be discontinued.

#### 4.4.3 Need for Further Training

Respondents were asked whether they believed that the training programme had provided them with the necessary competencies to handle the PCA job; 88% responded affirmatively. Additionally, 31% of respondents indicated a need for further training, whereas 43% did not, and 26% were unsure. PCAs were allowed to suggest changes or additions to the training programme. Several respondents had requests to extend the training programme's content to include a broader set of themes related to the role of a PCA: ‘The training related to respiratory care is totally fine. But even though you're really “just” required to handle this, there are a whole lot of other things to deal with. As PCAs, we are the ones closest to the user 24/7, and we can't distance ourselves from dealing with all sorts of psychological and personal matters around the user and the user's family’. Themes such as communication strategies, conflict management and disease‐specific knowledge were requested.

## 5 Discussion

The results of this study provide insights into the work‐life perceptions of Danish PCAs working in the HMV setting. Although PCAs predominately had positive perceptions of their work‐life, they also experienced varying kinds of challenges when working in the complex in‐home setting.

Although the quantitative results indicated overall satisfaction with communication among the various actors in the HMV setup, the qualitative findings revealed specific communicative challenges. Communication with the user was perceived as challenging when the user could not communicate sufficiently. Similar studies in the HMV field point to communication as an essential factor in sustaining employment, noting that limited communication with the user can be challenging and dissatisfying since the lack of conversation and feedback often leads to work‐life loneliness [28, 29, 39]. According to Winther et al. [13], family members of HMV users with ALS suggest that retaining and hiring PCAs becomes increasingly difficult as the disease progresses and communication challenges intensify.

Some PCAs felt unaccepted by the family, either due to a lack of communication from family members or because they experienced being judged negatively, which aligns with existing literature. Although the user and family are dependent on PCAs, they are also burdened by their presence [13]. Thus, some PCAs are expected to remain ‘invisible’ and are discouraged from interacting with the family, resulting in feelings of being unwanted [13, 24, 25, 26]. According to Dybwik et al. [29], PCAs find relationship formation and cooperation with the family to be the most challenging aspect of their job.

Some PCAs, particularly those employed through agencies, reported challenges in communication with their employer, expressed as both physical and figurative distance. They expressed a need for support; however, employment consultants often failed to respond to their inquiries. Several studies similarly find a considerable need for support when providing care in a home setting, along with experiences of insufficient communication and availability of support [30, 39, 40, 41]. Overall, PCAs found communication with the respiratory centre to be supportive, though some reported not feeling taken seriously when seeking assistance.

Regarding work satisfaction, the majority of PCAs expressed some measure of satisfaction with their job overall (83%), their physical work environment (75%) and their psychological work environment (69%). Working as a PCA was defined by general working conditions that some PCAs found favourable, whereas others did not, that is, working alone. Additionally, work satisfaction varied depending on the specific working conditions present in each individual home.

Some PCAs reported a poor physical work environment, including limited space and poor work postures, which aligns with similar studies that found the work environment to be poorly organised ergonomically [5, 7, 20]. Accordingly, some PCAs in the present study were concerned about being at risk of workplace injuries and felt that no one took responsibility for the physical work environment in the home setting. The psychological work environment was a prominent concern for many PCAs, who consistently expressed their needs in this area. PCAs described being mentally burdened by the significant responsibility of providing life‐sustaining respiratory care and monitoring. Additionally, some were negatively impacted by the user's challenging behaviour or their desire to support family members struggling with a difficult life situation. Thus, PCAs felt responsible for multiple aspects of the user and the family's life with HMV, which was a mental strain. This finding is consistent with existing literature describing how PCAs experience burnout [40] and stress due to excessive responsibilities [39]. Furthermore, the perceived lack of organisational support consequently made PCAs feel left to themselves in challenging situations, affecting their work environment negatively. This point is supported by several studies, describing PCAs as having no one to turn to for support [14, 30, 39], feeling overlooked and isolated [41], being disconnected from the broader patient care system and feeling forced to compensate for the lack of support through self‐management [42]. According to Dybwik et al. [29], PCAs have resigned due to the mental burden of working alone in challenging situations without adequate support. Similarly, Butler et al. [40] report that mental burnout among home care workers and a lack of support from their employment agency could cause them to resign.

Although the experiences of lack of support are well documented in the literature, the reasons behind this remain unexplained. Various factors may contribute to PCAs' perceptions of insufficient organisational support. HMV setups are inherently complex, catering to users with diverse diseases that vary in severity and prognosis, leading to differing care needs. Furthermore, HMV setups are complex due to their organisational structure, involving multiple formal actors, each with distinct responsibilities, services, separate interests and economic incentives. Although respiratory care and general personal care are often divided both organisationally and financially, these responsibilities are frequently performed by the same PCA. This organisational division of labour could be seen as fragmented and reductionist, conflicting with the comprehensive and holistic sense of responsibility expressed by PCAs.

According to Bolton et al. [43], complex and interdependent organisations, like HMV setups, require a high level of coordination between the involved formal actors to achieve the desired outcomes. The organisational theory of *Relational Coordination* proposes that relationships characterised by shared goals and knowledge, mutual respect and efficient communication enable effective work coordination in complex settings [43]. Applying this approach among formal actors in the HMV setup, such as the respiratory centre, the employer and the municipality, could help address the fragmented nature of HMV setups. By fostering continuous communication on shared goals and responsibilities, relational coordination could be strengthened at the organisational level, thereby improving the organisational support available to PCAS in delivering high‐quality HMV care.

Most PCAs believed that the training programme had provided them with the necessary competencies to handle respiratory care and monitoring. Still, 31% of respondents believed that they needed further training. Their needs encompassed a broader range of topics related to their overall role, including communication strategies, conflict management and disease‐specific knowledge. Other studies describe similar training needs among PCAs and home care workers, such as illness‐specific knowledge, understanding users' psychological needs and training in communication skills [30, 39, 41, 42]. These training needs align with the work‐life challenges experienced by PCAs and may reflect the holistic approach they wish to apply. In this study, 34% of respondents did not have a formal health education, and 32% had only 0–1 years of experience as a PCA. Thus, a substantial proportion of respondents had limited knowledge and experience to draw on. It can be assumed that training in the requested areas would better prepare PCAs to provide high‐quality care for HMV users and improve their ability to manage challenging situations when working independently in the home setting. This may even have the potential to support PCAs in effectively applying a PFCC approach [20] to HMV care, in line with the holistic sense of responsibility expressed.

**FIGURE 4:**
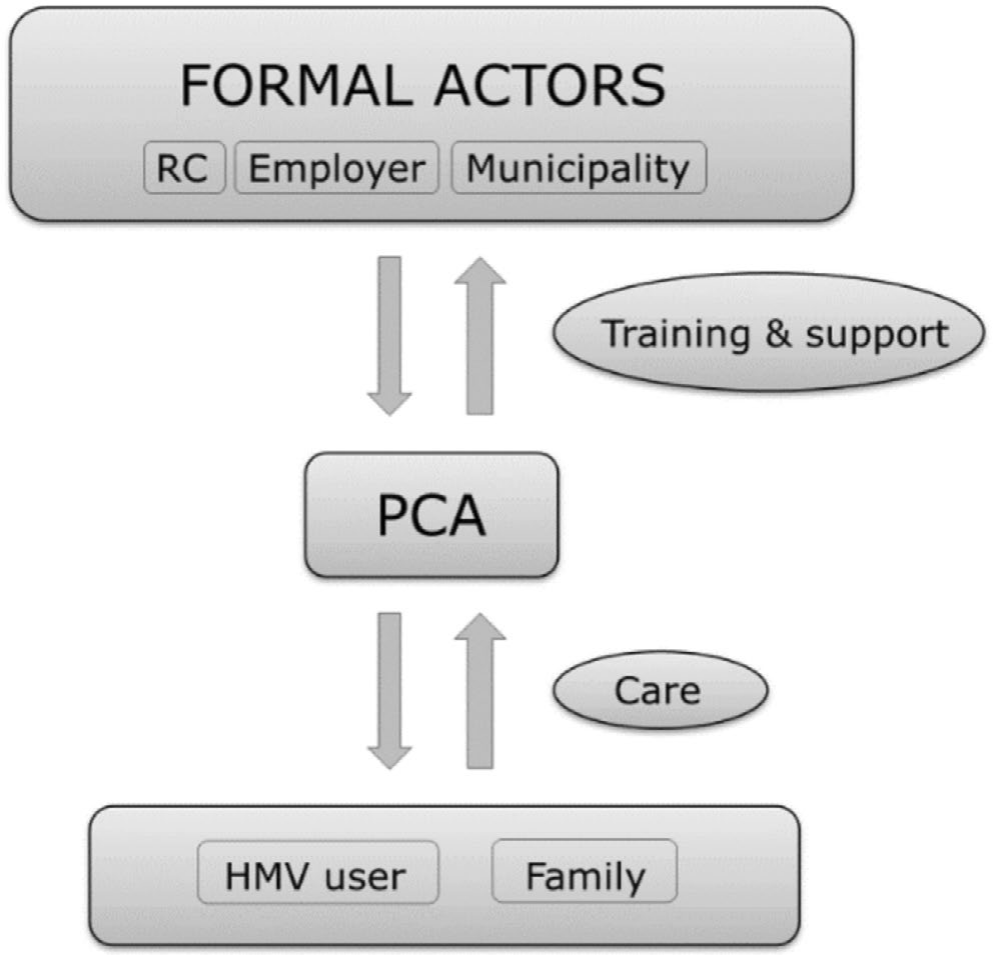
Interaction between actors in the HMV setup.

Although the quantitative results of this study suggest that PCAs are generally satisfied with their work‐life, the qualitative results revealed various challenges. This discrepancy may indicate significant variations in PCA experiences, which could be due to factors in the individual workplace, such as the complexity of the HMV user's needs, the severity of their diagnosis and family dynamics. The results indicate that ensuring health care quality for HMV users extends beyond individual formal actors prescribing and financing necessary services; it also requires providing PCAs with the necessary organisational support and training to perform these services, as illustrated in Figure 4. The work‐life challenges described in this study could potentially result in significant negative outcomes, such as poor staff retention and breakdown of PCA teams, affecting the quality of care for HMV users and their families. In this light, offering differentiated support and training may be beneficial.

## 6 Limitations

Using a web‐based descriptive survey design that included all PCAs registered in a registration system at the respiratory centre was a strength, as it allowed access to a broad group of Danish PCAs. Contacting PCAs directly via their personal email addresses rather than through their employers or the HMV users underscored that their responses would remain confidential, aiming to build trust and improve response rates. Studying PCAs collectively without distinguishing between subgroups, such as those working with children versus adults, may overlook specific characteristics related to these subgroups, highlighting the need for further investigation.

Methods triangulation was achieved by combining quantitative question options with free‐text questions, allowing qualitative descriptions. The substantial amount of qualitative data, illustrating PCAs' need to share their experiences, was addressed through qualitative content analysis. This analysis provided additional context for interpreting the descriptive data and added nuance on PCAs' perceived work‐life, which was considered a strength.

PCAs affiliated with the respiratory centre were not registered in the system until 2019, making it impossible to include PCAs employed before that year. The exact number of excluded PCAs is unknown, but the total population of PCAs at the respiratory centre was estimated to be 2–2500 at the time of the study. This exclusion could negatively affect representativeness, as it could be assumed that some PCAs employed before 2019 would have more extensive experience. Thus, using the registration system for sampling and distribution was subject to some uncertainty, which was a study limitation.

The considerable number of lost respondents may also be attributed to the uncertainties in using the registration system. Since PCAs are not systematically deregistered upon resignation, the actual number of PCAs at the time of the study could be lower, leading to a falsely low response rate. This is not unlikely, given the documented high turnover rate of PCAs [15]. Although the unclear response rate is a clear limitation, the substantial amount of qualitative data ensures the relevance of the overall results. Repeating the study as a multicenter study and using updated registration technologies to improve response rates could be relevant.

## 7 Conclusion

This study showed that the heterogeneous group of PCAs working with HMV generally rated their work‐life positively; however, they also faced challenges in various areas of their work‐life. A key contributing factor was the complexity of their work, involving communication and collaboration with HMV users and their families facing physical and mental challenges. An important contribution of this study is the understanding that taking on a holistic sense of responsibility for the user and family's entire life situation independently placed a considerable mental burden on PCAs, negatively affecting their work environment. A critical factor identified in relation to this was the lack of communication and organisational support from formal actors within the HMV setup, leaving PCAs feeling left to themselves. PCAs advocated for a more comprehensive training programme to adequately address the full scope of complex care needs of the HMV user and their family. Thus, PCAs were dedicated to providing holistic and high‐quality HMV care but emphasised the essential need for improved organisational support and training to effectively navigate the diverse and complex situations encountered in the home setting. These results contribute new and important insights for clinical practice regarding the complex nature of PCAs' work‐life experiences and the barriers encountered in providing high‐quality HMV care. They call for improved communication, support and training within HMV setups, ideally informed by organisational research on coordination at the HMV setup level, to gain a deeper understanding of the mechanisms underlying the results of this study.

## Author Contributions

The first author was responsible for drafting the manuscript and contributed most of the writing and analysis. The other authors were responsible for the study conception, data collection and review and editing of the manuscript.

## Ethics Statement

According to Danish law, questionnaire studies of this type do not require formal ethical approval.

## Conflicts of Interest

The authors declare no conflicts of interest.

## Supporting information


**Appendix S1:** scs70098‐sup‐0001‐AppendixS1.docx.

## Data Availability

The authors have nothing to report.
